# Halal Food Performance and Its Influence on Patron Retention Process at Tourism Destination

**DOI:** 10.3390/ijerph18063034

**Published:** 2021-03-16

**Authors:** Heesup Han, Linda Heejung Lho, António Raposo, Aleksandar Radic, Abdul Hafaz Ngah

**Affiliations:** 1 College of Hospitality and Tourism Management, Sejong University, 98 Gunja-Dong, Gwangjin-Gu, Seoul 143-747, Korea; heesup.han@gmail.com (H.H.); heeelho@gmail.com (L.H.L.); 2 CBIOS (Research Center for Biosciences and Health Technologies), Universidade Lusófona de Humanidades e Tecnologias, Campo Grande 376, 1749-024 Lisboa, Portugal; 3 Independent Researcher, Gornji Kono 8, 20000 Dubrovnik, Croatia; aleradic@gmail.com; 4 Faculty of Business, Economy and Social Development, Universiti Malaysia Terengganu, Kuala Nerus 21030, Terengganu, Malaysia; hafaz.ngah@umt.edu.my

**Keywords:** halal food performance, availability, healthy/nutritional factor, accreditation, clean/safe/hygiene factor, trust, attachment, halal-friendly image, retention, muslim travelers

## Abstract

Muslim tourism is one of the most rapidly developing sectors in the international tourism industry. Nevertheless, halal food performance and its relationship with international Muslim traveler decision-making and behaviors have not been sufficiently examined. The present research explored the influence of halal food performance, which encompasses availability, health/nutrition, accreditation, and cleanness/safety/hygiene factors, on the Muslim traveler retention process at a non-Islamic destination. A survey methodology with a quantitative data analytic approach was employed to achieve research goals. Our findings indicated that halal food performance increased destination trust and destination attachment, which in turn influenced Muslim traveler retention. Additionally, the efficacy of the higher-order framework of halal food performance was defined. Both destination trust and attachment mediated the effect of halal food performance on retention. A halal-friendly destination image included a moderating influence on the retention process. The effectiveness of the proposed theoretical framework for explicating Muslim traveler behaviors was uncovered. This research better introduces the importance of halal food performance and its attributes for the elicitation of Muslim traveler approach responses and behaviors at a non-Islamic destination to researchers and practitioners.

## 1. Introduction

Muslim travelers are undoubtedly an emerging tourist group in the global tourism industry [1,2,3,4,5]. As the competition in the international tourism marketplace is intensifying, many destinations are eager to develop halal-friendly products and make a Muslim-friendly tourism environment to attract a greater number of Muslim visitors [1,4]. Developing new halal-friendly marketing and retention strategies is irrefutably important in expanding the business volume related to Muslim tourism at tourist destinations [1,5,6]. Halal food is frequently regarded as one of the most significant halal-friendly products at many places [7,8,9,10]. Increasing the halal food availability and improving its attribute quality is indisputably becoming essential to fulfill Muslim travelers’ halal-friendly tourism needs and provide them with pleasant tourism experiences, especially in non-Islamic countries and tourism destinations [10,11].

Despite the rapid growth of Muslim tourism for the past few decades in the global tourism sector, the competitiveness with many non-Islamic destinations in the marketplace is still not strong enough [4,5,10]. According to [12], such weak competitiveness is mostly relevant to Muslim travelers’ experiences with restaurants and foods that are not sufficiently halal-friendly. Nevertheless, the empirical endeavor investigating the possible influence of halal food performance on Muslim traveler post-purchase behavior at non-Islamic destinations has hardly been made. In addition, the existing studies indicated the criticality of trust and attachment in explicating the traveler retention process [13,14,15,16]. Scant research has unearthed the possible linkages between halal food performance and these essential concepts. Moreover, destination image is irrefutably a crucial variable, affecting the entire traveler post-purchase decision-making procedure [17,18,19]. A halal-friendly destination image is vital in Muslim traveler behaviors [20]. Yet, how a halal-friendly destination image determines the magnitude of the relation strength between halal food performance and its outcome constructs has hardly been examined. That is, the particular role of a halal-friendly destination image as a moderator in the Muslim visitor retention process has not been thoroughly explored.

Taking this into account, the current research was devised to build a theoretical framework that explicates Muslim traveler retention formation in a clear manner. Many previous studies focused on defining what halal food is and what halal tourism is. Thus, the current research tried to take a step further from the latent literature and develop both the theoretical and the practical contribution of halal food to halal tourism. In particular, we aimed (1) to assess the possible impact of halal food performance, which comprises availability, health/nutrition, accreditation, and cleanness/safety/hygiene factors as its constituents, on the Muslim visitor retention process at a non-Islamic destination; (2) to uncover the appropriateness of a higher-order structure of halal food performance and its competence; (3) to investigate the convoluted relations among halal food performance, destination trust, and destination attachment, and the possible influence on retention; (4) to assess the mediating role of destination trust and attachment; and (5) to unearth the moderating role of a halal-friendly destination image within the proposed conceptual framework. The remaining parts of this research are the literature review, methodology, data analysis and results, and discussion and implications. Investigating these five aims, this research will be able to not only build a theoretical framework of Muslim traveler retention but also discover practical contributions for ways to retain Muslim travelers.

## 2. Literature Review

### 2.1. Halal Food and Its Performance

Halal is an Arabic term indicating, “which is allowed by Islamic teaching” [9,11]. Halal dietary laws were developed within Asia for Islamic followers to practice a nutritious and healthy lifestyle [21]. The foods permitted under these halal dietary laws are called halal food [21]. Muslim travelers’ demand for overseas traveling is rapidly increasing, and their primary distress is the accessibility and quality of halal food in an international destination [1,9,11]. Indeed, [22] recently reported that halal food consumption is a key aspect of Muslim tourism. Little availability (or unavailability) and low performance of halal food at a destination lowers the quality of Muslims’ overall tourism experiences and generates their avoidance behaviors for that place [2,10]. Due to the recent growth of the Muslim tourism market [7,23], halal food is becoming a fundamental topic in the international tourism industry [8,11,20].

According to [24], the term “performance” indicates that individuals’ evaluation of the excellence of a product and its attributes as compared to other products available in the marketplace offered by rival firms. Consistently, halal food performance in this research refers to travelers’ appraisal of the excellence of halal food and its essential attributes as compared to the halal foods offered by competing destinations. Halal food performance is constructed as an amalgamation of multiple dimensions that explicate an intricate aspect of food and beverage consumption in a halal-friendly way [11,12]. Halal food availability factors, halal food health/nutrition factors, halal food accreditation factors (e.g., accreditation with a halal certificate, halal logos), and halal food cleanness/safety/hygiene factors are all crucial constituents of assessing halal food performance at international destinations [9,20,25]. Indeed, [12] and [11] asserted that availability, health/nutrition, accreditation, and cleanness/safety/hygiene are the main things for Muslims to consider related to halal food consumption when planning and practicing halal-friendly tourism activities at non-Islamic destinations.

Research on halal food and understanding its influence on Muslim traveler behaviors is still in its infancy in many non-Islamic destinations [1,6]. In these destinations, halal food and beverage are relatively a new type of tourism product [11,26]. Muslim travelers can be somewhat skeptical about whether the foods provided in non-Islamic destinations are pure halal [11]. Although the authenticity and decency of halal food in such destinations is not entirely certain [27], the quality performance of halal food often inspires Muslim travelers’ high level of trust for the food and the places [1,12], elicits their affection and attachment to the places [8,20], and affects approach behaviors for the destinations [2,28]. Simply put, halal food and its performance are strongly relevant to responses and behaviors among overseas Muslim travelers [8,28].

### 2.2. Destination Trust

Because of the intangible form of hospitality and tourism products, trust and reliability of the products/services often become an important subject in the hospitality and tourism marketplace [14,15]. Many hospitality/tourism products entail a certain level of uncertainty/risk, particularly in the international tourism context [14,29,30]. While the definition of trust varies in the existing literature, one of the most broadly accepted definitions in tourism is that trust is the degree to which travelers rely on a product, place, brand, or exchanging partner [15]. Similarly, [31] conceptualized destination trust as the travelers’ level of confidence and reliability toward a tourist destination and its performance. It is broadly known that trust is a critical influence factor on travelers’ emotional attachment and approach behaviors [13,15,29,31]. Thus, destination trust will positively affect destination attachment.

For instance, in consumer behavior, [32] found that trust is an essential driver of repurchase intention. In addition, [15] uncovered that trust in a place helps visitors attach to the place and plays a vital role in building their positive intentions for the place in the international tourism context. In recent decades, the importance of trust has been especially emphasized in the Muslim tourism/consumer behavior sector [10,13,14]. Such trust is likely formed based on a Muslim-friendly tourism environment at the destination [10]. In the Muslim tourism context, trust also generates travelers’ emotional responses and reactions that are favorable for tourism destinations [28,33]. In other words, destination trust will positively affect Muslim traveler retention.

### 2.3. Destination Attachment

A comprehension of attachment has a meaningful implication for understanding traveler behavior [16,34,35,36]. Due to its hefty contribution to a surge of positive decision/behavior of visitors, attachment to a destination/place has broadly been a crucial concept in hospitality and tourism [36]. Generating such attachment can be described as the process of building an emotional connection between an individual and a destination [16]. Coherently, destination attachment in the present research indicates Muslim travelers’ emotional ties to a specific tourism destination. Attachment plays a crucial role when individuals choose tourism/leisure products [34,36,37]. Findings in the extant literature demonstrated that the high tourist attachment to a tourism product/service/destination affects his/her post-purchase decision formation, inducing positive behaviors for the product/service/destination [35,38].

In the hospitality context, [39] explored the influence of patrons’ attachment/involvement. In [38], the findings revealed that the patrons’ purchase decision formation for a hospitality product is significantly impacted by their level of product attachment. In the cruise tourism sector, [35] investigated the intricate process of passenger loyalty generation. Their empirical finding showed that passenger loyalty formation is significantly triggered by passenger attachment to the cruise line. In the festival tourism sector, [36] discovered that visitors’ attachment to a festival destination is significantly associated with their loyalty for the place. More recently, [37] uncovered that travelers’ level of attachment to the tourism product/place increases their approach behaviors, such as retention and word-of-mouth activities. Travelers’ strong attachment to a destination boosts their willingness to revisit the place, whereas travelers’ weak attachment increases their avoidance decisions [16,36]. Therefore, destination attachment will positively affect Muslim traveler retention.

### 2.4. Halal-Friendly Destination Image

Image has long been believed a crucial dimension when explaining patrons’ decision-making procedures and attitudes of consumption [17,18,39,40]. Image refers to patrons’ overall perception of a firm and its product(s) based on the relations held in their memory [41]. Kotler et al. [42] provided a more precise conceptualization of it where image is the overall set of consumers’ perceptions, opinions, and beliefs about a firm and its product(s). Coherently, in this research, destination image indicates the summation of perceptions, opinions, and beliefs that Muslim travelers have about a place and its halal-friendly attributes. Individuals’ impressions/thoughts/beliefs of a particular object are formed ultimately based on attained and processed information [43]. Baloglu, McCleary [44] and Lee et al. [45] thus indicated that building an image of a place/product in the consumer behavior context is a subjective and cognitive process of assessing such information about a place/product.

Image and its potential influence have been well documented in consumer behavior and tourism literature [19,39,45]. It is widely believed that increasing an image of a firm/place eventually results in increased repeat business and loyalty for the firm/place [40,44,46]. According to [47], one’s image of a place significantly impacts the formation of his/her revisit intention in the hospitality sector. More recently, [19] uncovered that destination image among travelers’ social network members has a considerable effect on their choice behaviors. It is likely that the entire customer retention process becomes strong when a customer image of a place/product is favorable, whereas said process becomes weak when he/she has an unfavorable image of the place/product [18,40,45]. Likewise, while a positive image of a place/product often reinforces the formation of patrons’ post-purchase decisions, a negative image often weakens their decision formation [18,39,47]. It is also evident that travelers’ image, which is positive for a particular destination/tourism product, considerably influences the process of generating their favorable behavioral intentions toward the destination/tourism product [45]. Consequently, it can be concluded that a halal-friendly destination image includes a significant effect on the relation between halal food performance and destination trust and the relation between halal food performance and destination attachment.

### 2.5. Proposed Model and Hypothesis

**Figure 1:**
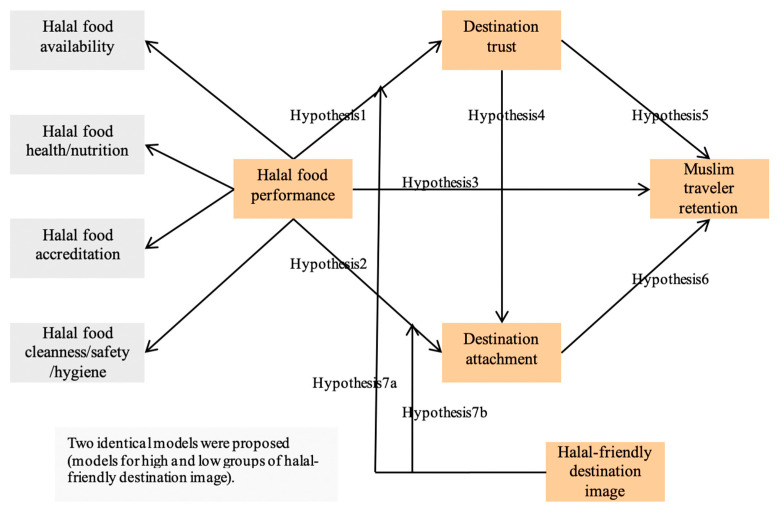
The proposed conceptual framework. H1: Halal food performance includes a positive effect on destination trust. H2: Halal food performance includes a positive effect on destination attachment. H3: Halal food performance includes a positive effect on Muslim traveler retention. H4: Destination trust includes a positive effect on destination attachment. H5: Destination trust includes a positive effect on Muslim traveler retention. H6: Destination attachment includes a positive effect on Muslim traveler retention. H7a: Halal-friendly destination image includes a significant effect on the relation between halal food performance and destination trust. H7b: Halal-friendly destination image includes a significant effect on the relation between halal food performance and destination attachment.

The proposed model shown in Figure 1 encompassed (a) halal food performance whose first-order factors are availability, health/nutrition, accreditation, and cleanness/safety/hygiene; (b) destination trust; (c) destination attachment; (d) halal-friendly destination image; and (e) Muslim traveler retention. The model contained a total of seven research hypotheses. Hypotheses 1–6 concern the causal relationships among research constructs. In addition, Hypotheses 7a–7b pertain to the moderating impact of a halal-friendly destination image.

## 3. Method

### Measures

The measurement items for the research factors used in this research were adopted from the existing studies [11,12,16,17,29,48,49] and adjusted to be appropriate to the current study context. All items were measured by a seven-point Likert scale. Additionally, multiple items were used to assess each construct. A total of six items were adopted to evaluate halal food performance. Specifically, two items for halal food availability (e.g., “Availability of halal food attracted me to visit tourist places”), two items for halal food health/nutrition (e.g., “Halal foods served in restaurants are healthy and nutritious”), two items for halal food accreditation (e.g., “Halal food providers in tourist sites are accredited with halal certification”), and two items for halal food cleanness/safety/hygiene (e.g., “Halal food and beverage offered in tourist sites/places were clean, safe, and hygienic”) were utilized.

To measure destination trust, three items were used (e.g., “I have confidence in Korea as a halal-friendly destination”). Destination attachment was assessed with two items (e.g., “I feel emotionally attached to Korea as a tourist destination”). Additionally, we utilized three items to assess a halal-friendly destination image (e.g., “Overall, I have a good image of Korea as a Halal-friendly destination”). For assessing Muslim traveler retention, two items were utilized (e.g., “Korea as a Halal family-friendly place will be my first choice when it comes to choosing a destination”). The survey questionnaire comprising these measures was pre-tested with tourism researchers. A minor modification was adopted based on their feedback. The survey was then further modified by halal tourism specialists’ reviews. All measures used in this research are exhibited in Appendix A.

## 4. Results

### 4.1. Data Collection and Sample Profiles

A field survey was conducted. The survey was carried out in many tourist places of Korea, which include restaurants, hotels, shopping malls, cultural districts, and tourist sites. Many international travelers prefer these places. The surveyors contacted Muslim tourists and asked if they were willing to fill out the survey. All survey participation was voluntary. Detailed information about the survey and its objectives were explained to all participants. The participants were asked to read the mandatory survey instructions thoroughly and answer the questions. All responses were returned onsite. Throughout this process, the researchers attained a total of 326 usable responses, which were used to analyze the data. Of 326 participants, 54.3% were female tourists, whereas 45.7% were male tourists. The participants’ age range was 20–69 years old. The average age was 29.17 years old. When visit frequency was questioned, roughly 65.6% showed that they had visited Korea only once. About 89.3% indicated that they had visited Korea three times or less. A majority of the respondents classified themselves as pleasure travelers (55.2%), then education travelers (36.2%), and lastly business travelers/others (8.6%). About 77.9% indicated that their annual income is less than USD 39,999 (77.9%), followed by between USD 40,000 and USD 99,999 (20.3%), and then USD 100,000 or higher (1.8%). The sample respondents were, in general, highly educated. About 55.2% indicated they are college graduates, then graduate degree holders (28.8%), and then high school graduates or less (16.0%). Asking the following question, “For you, how important are halal-friendly food products when choosing a destination,” most survey participants reported that halal foods are important or very important when selecting a tourism destination (87.1%).

### 4.2. Confirmatory Factor Analysis and Measurement Model Evaluation

A confirmatory factor analysis was performed (shown in Table 1). The measurement model contained a satisfactory level of goodness-of-fit statistics (*χ^2^* = 318.108, df = 107, *p* < 0.001, *χ^2^*/df = 2.973, RMSEA = 0.078, CFI = 0.958, IFI = 0.958, TLI = 0.939). Each loading value was found significant at *p* < 0.01. A composite reliability test was conducted. All reliability values (halal food availability = 0.785, halal food health/nutrition = 0.903, halal food accreditation = 0.833, halal food cleanness/safety/hygiene = 0.737, destination trust = 0.945, destination attachment = 0.829, halal-friendly destination image = 0.949, and Muslim traveler retention = 0.732) were above the suggested threshold of 0.70 [50]. Therefore, the internal consistency of the construct measures was apparent. Additionally, the calculated average extracted values (halal food availability = 0.647, halal food health/nutrition = 0.824, halal food accreditation = 0.713, halal food cleanness/safety/hygiene = 0.584, destination trust = 0.852, destination attachment = 0.707, halal-friendly destination image = 0.861, and Muslim traveler retention = 0.579) were all higher than the recommended level of 0.50 [50]. Moreover, these values were higher than the correlations (squared) between factors (refer to Table 2). Hence, construct validity (convergent and discriminant) was evident in this research.

### 4.3. Structural Model Analysis and Hypotheses Testing

**Figure 2:**
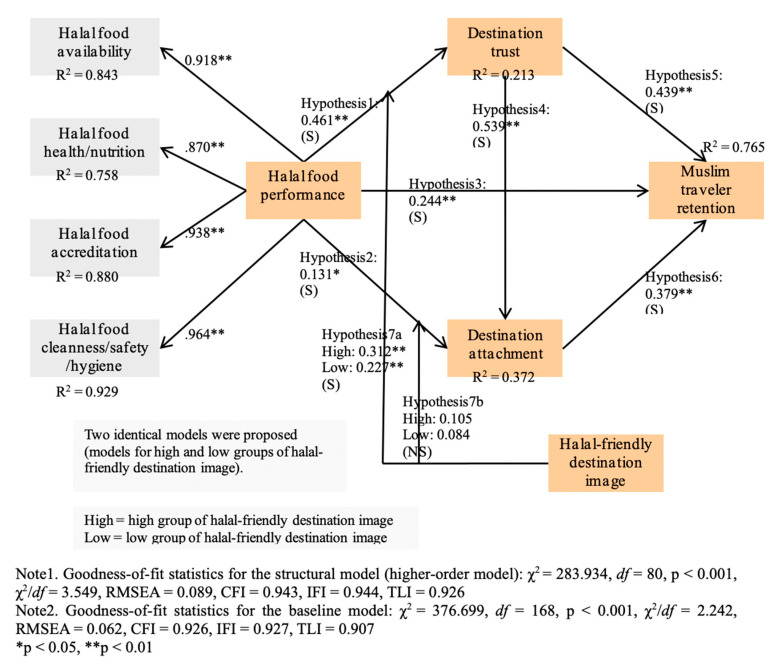
The structural model results.

A structural equation modeling was performed. The proposed model was found to have an acceptable level of goodness-of-fit statistics (χ^2^ = 283.934, df = 80, *p* < 0.001, χ^2^/df = 3.549, RMSEA = 0.089, CFI = 0.943, IFI = 0.944, TLI = 0.926). The details are exhibited in Table 3 and Figure 2. As exhibited in Figure 2, the higher-order model for halal food performance result indicated that the first-order dimensions (availability, health/nutrition, accreditation, and cleanness/safety/hygiene) and the higher-order latent construct are related in a significant manner (*p* < 0.01). Coefficient values (standardized) for such relationships were 0.918 (availability), 0.870 (health/nutrition), 0.938 (accreditation), and 0.964 (cleanness/safety/hygiene), correspondingly. The relations were all significant (*p* < 0.01). The first-order dimensions of availability (R^2^ = 0.843), health/nutrition (R^2^ = 0.758), accreditation (R^2^ = 0.880), and cleanness/safety/hygiene (R^2^ = 0.929) were sufficiently alleged to its global latent factor. The framework encompassing halal food performance dimensions as direct drivers of its outcome variables (the first-order formative research model) was run to compare it to the proposed higher-order model. However, the result indicated that most halal food performance factors within the first-order formative research model were not significantly associated with destination trust, destination attachment, and retention. Therefore, it was clear that the first-order variables related to one global factor of halal food performance.

The proposed associations were tested. As expected, halal food performance exercised a significant influence on destination trust (*β* = 0.461, *p* < 0.01), destination attachment (*β* = 0.131, *p* < 0.05), and Muslim traveler retention (*β* = 0.244, *p* < 0.01). This outcome supported Hypotheses 1, 2, and 3. The hypothesized influence of destination trust was evaluated. Our finding showed the significant linkages between destination trust and destination attachment (*β* = 0.539, *p* < 0.01) and between destination trust and Muslim traveler retention (*β* = 0.439, *p* < 0.01). Hence, Hypotheses 4 and 5 were found true. In addition, as anticipated, destination attachment was a significant predictor of Muslim traveler retention. Hence, Hypothesis 6 was supported (*β* = 0.379, *p* < 0.01). Muslim traveler retention was satisfactorily accounted for by its antecedents (R^2^ = 0.765). Moreover, about 37.2% and 21.3% of the total variance in destination attachment and destination trust were described by their predictors, respectively.

The indirect influence of research constructs was observed. As shown in Table 2, our result showed that trust significantly affected retention indirectly through destination attachment (*β* = 0.204, *p* < 0.01). In addition, halal food performance encompassed a significant indirect impact on destination attachment (*β* = 0.248, *p* < 0.01) and Muslim traveler retention (*β* = 0.346, *p* < 0.01). This finding indicated that both destination trust and attachment played a critical mediating role within the proposed model. Our further investigation showed that destination trust had the strongest total impact on Muslim traveler retention (*β* = 0.643, *p* < 0.01), halal food performance (*β* = 0.590, *p* < 0.01), and then destination attachment (*β* = 0.379, *p* < 0.01).

### 4.4. Baseline and Structural Invariance Model Results

A metric invariance test was carried out to uncover the proposed effect of a halal-friendly destination image. The obtained responses were split into high and low image groups. The high group contained 151 cases, whereas the low group included 175 cases. This group was completed on the basis of the K-means cluster analysis result. A baseline model comprising these two groups was created. The baseline model assessment results are shown in Table 4. The model contains a satisfactory level of goodness-of-fit statistics (χ^2^ = 376.699, *df* = 168, *p* < 0.001, χ^2^/*df* = 2.242, RMSEA = 0.062, CFI = 0.926, IFI = 0.927, TLI = 0.907). Within the model, all loadings were constrained to be equivalent across groups. We conducted a Chi-square test subsequently.

The result of the comparison between the baseline model and the constrained model indicated that the path from halal food performance to trust was meaningfully different between the high and low groups of halal-friendly destination image (Δχ^2^ [1] = 4.164, *p* < 0.05). This result supported Hypothesis 7a. However, the linkages from halal food performance to attachment (Δχ^2^ [1] = 0.286, *p* > 0.05) were not meaningfully different between the high and low groups. Thus, Hypothesis 7b was rejected. Figure 2 and Table 3 include the specifics about the metric invariance test results.

## 5. Discussion

### 5.1. Higher-Order Framework of Halal Food Performance and Implications

One of the meaningful points in the present research is the higher-order framework of halal food performance. The four first-order variables, such as (1) availability, (2) health/nutrition, (3) accreditation, and (4) cleanness/safety/hygiene, belong to one inclusive latent factor of halal food performance. This means that the commonality underlying the four first-order variables was wholly extracted by its second-order construct. This empirical result and finding enrich the halal food literature by providing a hierarchical approach, which clearly apprehends the halal food performance. The parsimonious higher-order structure framework enlightens academics and practitioners about the competence of theorizing intricate halal food performance factors in a more succinct manner in the Muslim tourism sector.

Food cleanness/safety/hygiene and accreditation were two main factors of halal food performance. Therefore, for practitioners, assuring that halal foods served in restaurants and available in tourist sites are clean, safe, and hygienic is critical. Displaying a halal logo clearly and providing halal certified/accredited food/service is also essential. Such efforts would fulfill many crucial facets of overseas Muslim travelers’ needs. In addition, food availability and health/nutrition were the other crucial dimensions of halal food performance. Boosting the availability of halal food and enhancing the healthy and nutritious facets of existing halal food are hence imperative for a non-Islamic destination product to appeal to Muslim visitors.

More than 20.0% of the food industry in the world is related to halal foods, and its volume is increasing with the constant growth of the international Muslim traveler population [20]. Many non-Muslim countries can also use halal foods as a tactic of their Muslim tourism development. As demonstrated in this research, halal food performance ultimately results in Muslim traveler retention. Improving the performance of halal food by centering on its cleanness/safety/hygiene, accreditation, availability, and health/nutrition can elicit approach behaviors for destinations, eventually increasing business opportunities, creating more jobs, and bringing monetary investment/benefit to the destinations. Theoretically, this research successfully explored halal food performance and its potential influence, which was weakly known. Accordingly, diverse aspects of halal food performance should be actively utilized when developing/building a theoretical framework for explicating international Muslim traveler responses and behaviors at non-Muslim destinations.

### 5.2. Implications Related to the Role of Destination Trust and Attachment

Prior research in the literature asserted that the influence of cognitive variable(s) on traveler post-purchase behaviors is likely to be strengthened by the mediating effect of trust or attachment [12,29,30,37]. In line with these studies, the present research finding demonstrated that destination trust and destination attachment significantly mediated the impact of halal food performance on Muslim traveler retention. This means that both destination trust and destination attachment acted as intensifiers of Muslim traveler retention within our proposed theoretical framework. Our results offer tourism academics and destination practitioners crucial information about the importance of escalating destination trust and enhancing destination attachment in order to induce the maximum impact of halal food performance on Muslim visitor retention. Given the elaborate theoretical mediating mechanism unearthed in the present research, it is imperative to deal with such mediator variables for the effectual increase in Muslim visitor approach behaviors for a non-Islamic tourism destination.

### 5.3. Moderating Effect of Halal-Friendly Destination Image and its Implications

Findings of our metric invariance test indicated that the relationship between halal food performance and destination trust is moderated by a halal-friendly destination image. In particular, the association strength was greater in the high group of destination image than in the low group (high group: *β* = 0.312, *p* < 0.01 vs. low group: *β* = 0.227, *p* < 0.01). This result implies that at a similar level of halal food performance, Muslim visitors who have a strong halal-friendly image of a non-Islamic location more heavily rely on the destination and its tourism environment than those who have an unfriendly image. Theoretically, this finding has a strong value as the present study is the first empirical research that provides evidence regarding the significance of a halal-friendly destination image in determining the magnitude of the influence of halal food performance on destination trust. Our evaluation and finding of the convoluted associations among food performance, destination image, and destination trust, which are especially crucial in the international Muslim tourism context, contribute to escalating academics’ understanding of Muslim visitors’ post-purchase formation regarding non-Islamic destination products. Our finding is also practically meaningful. As a halal-friendly image about a destination is decisive in Muslim visitors’ retention process, practitioners should be more aware of its criticality. Offering superior services and developing/providing new products, which are entirely friendly for Muslim visitors, could generate a positive halal-friendly destination image.

## 6. Conclusions and Limitations

### 6.1. Conclusions

The rapid growth of Muslim tourism is evident in many Islamic and non-Islamic destinations [4,5,10]. Yet, our understanding of halal food performance and its influence on Muslim traveler approach behaviors for a destination was lacking. The present research has filled this gap. The hypothesized conceptual framework was wholly supported. Halal food performance was revealed to be a determinant of destination trust, destination attachment, and Muslim traveler retention. Destination trust and attachment contributed to making the best use of halal food performance in the retention process. A Halal-friendly destination image strengthened the influence of halal food performance on destination trust. Our findings help practitioners at non-Islamic destinations invent useful strategies to retain international Muslim visitors by utilizing halal food, trust, attachment, and image as important tools. Indubitably, a theoretical base pertinent to Muslim traveler behaviors at non-Islamic places is in the infant stage. From this perspective, our research that helps academics and practitioners increase their understanding of such behaviors contains high originality and value.

### 6.2. Limitations and Future Research Arena

Although these research findings have presented both theoretical and conceptual frameworks, this research had some limitations that provide some directions for potential studies. First, the present study centered on Muslim traveler behaviors at a non-Islamic destination. Their behaviors can possibly differ at an Islamic destination. Future research should conduct an empirical comparison of the Muslim traveler retention process and behavior across Islamic and non-Islamic destinations, which would be a meaningful extension of this study. Second, it would be true that a normative process such as social norms and moral/ethical obligation is also crucial when explicating Muslim traveler approach or avoidance behaviors. Indeed, some research denoted the criticality of normative factors in traveler behaviors [51]. Thus, future research should broaden the proposed conceptual model by integrating normative influence in order to improve the comprehensiveness and explanatory power of the model. Lastly, future research should consider the changes in travel options. For example, the accommodation option of Airbnb and/or other rental properties will allow travelers to cook their own meals during their travel so that they do not have to consider the halal-friendly options.

## Figures and Tables

**Table 1:** A confirmatory factor analysis of measurement items (*n* = 326).

Measurement Items (Factor Loadings)	CR	AVE
HA1: Availability of halal food attracted me to visit tourist places. (0.739)	0.785	0.647
HA2: Halal food and beverage are served in restaurants and outlets in tourist sites/places. (0.865)		
HN1: Halal foods served in restaurants are healthy and nutritious. (0.921)	0.903	0.715
HN2: Halal foods that are available in tourist sites/places are healthy and nutritious. (0.894)		
HAC1: Halal food providers in tourist sites are accredited with halal certification. (0.843)	0.833	0.713
HAC2: Halal food outlets/restaurants in tourist sites clearly display a halal logo. (0.846)		
HC1: Halal food and beverage offered in tourist sites/places were clean, safe, and hygienic. (0.797)	0.737	0.584
HC2: Halal foods served in restaurants are clean, safe, and hygienic. (0.0.73)		
DT1: I think Korea as a halal-friendly destination is reliable. (0.901)	0.945	0.852
DT2: I have confidence in Korea as a halal-friendly destination. (0.963)		
DT3: I think that Korea as a halal-friendly destination has high integrity. (0.904)		
DA1: I like Korea more than other tourist destinations. (0.844)	0.829	0.707
DA2: I feel emotionally attached to Korea as a tourist destination. (0.838)		
HD1: My overall image of Korea as a Halal friendly destination is positive. (0.914)	0.949	0.861
HD2: My overall image of Korea as a Halal friendly destination is positive. (0.951)		
HD3: Overall, I have a good image of Korea as a Halal-friendly destination. (0.918)		
MT1: I am willing to revisit Korea in the near future. (0.686)	0.732	0.579
MT2: Korea as a Halal family-friendly place will be my first choice when it comes to choosing a destination. (0.829)		

Note. HA: Halal food availability, HN: Halal food nutrition, HAC: Halal food accreditation, HC: Halal food cleanness, DT: Destination trust, DA: Destination attachment, HD: Halal friendly destination trust, MT: Muslim traveler retention.

**Table 2:** Measurement model and data quality assessment results (*n* = 326).

Constructs	(1)	(2)	(3)	(4)	(5)	(6)	(7)	(8)
(1) Halal food availability	1.000	–	–	–	–	–	–	–
(2) Halal food health/nutrition	0.715 ^a^	1.000	–	–	–	–	–	–
	(0.511) ^b^							
(3) Halal food accreditation	0.659	0.715	1.000	–	–	–	–	–
	(0.434)	(0.511)						
(4) Halal food cleanness/safety/hygiene	0.653	0.628	0.751	1.000	–	–	–	–
	(0.426)	(0.394)	(0.564)					
(5) Destination trust	0.418	0.380	0.315	0.454	1.000	–	–	–
	(0.175)	(0.144)	(0.099)	(0.206)				
(6) Destination attachment	0.306	0.273	0.262	0.320	0.531	1.000	–	–
	(0.094)	(0.075)	(0.069)	(0.102)	(0.282)			
(7) Halal-friendly destination image	0.506	0.486	0.424	0.556	0.699	0.544	1.000	–
	(0.256)	(0.235)	(0.180)	(0.309)	(0.487)	(0.296)		
(8) Muslim traveler retention	0.465	0.406	0.370	0.470	0.654	0.585	0.759	1.000
	(0.216)	(0.165)	(0.137)	(0.221)	(0.428)	(0.342)	(0.576)	
Mean	4.824	4.399	4.747	4.848	4.405	4.704	4.432	4.432
Standard deviation	1.582	1.594	1.552	1.381	1.349	1.206	1.206	1.387

Note. Goodness-of-fit statistics: χ^2^ = 318.108, *df* = 107, *p* < 0.001, χ^2^/*df* = 2.973, RMSEA = 0.078, CFI = 0.958, IFI = 0.958, TLI = 0.939. ^a^ Between-construct correlations are below the diagonal. ^b^ Between-construct correlations (squared) are within parentheses.

**Table 3:** Structural equation modeling results and hypotheses testing (*n* = 326).

Hypothesized Paths				Standardized Estimates	*t*-Values
H1	Halal food performance		Destination trust	0.461 **	7.799
H2	Halal food performance	→	Destination attachment	0.131 *	2.091
H3	Halal food performance	→	Muslim traveler retention	0.244 **	4.359
H4	Destination trust	→	Destination attachment	0.539 **	8.461
H5	Destination trust	→	Muslim traveler retention	0.439 **	6.547
H6	Destination attachment	→	Muslim traveler retention	0.379 **	4.884
	Halal food performance		Halal food availability	0.918 **	-
	Halal food performance		Halal food health/nutrition	0.870 **	14.754
	Halal food performance		Halal food accreditation	0.938 **	13.774
	Halal food performance		Halal food cleanness/safety/hygiene	0.964 **	12.606
Total variance explained:		Indirect impact on retention:		Total impact on RI:	
R^2^ for Muslim traveler retention = 0.765		β _trust – attachment – retention_ = 0.204 **		β _destination attachment_ = 0.379 **	
R^2^ for destination attachment = 0.372					
R^2^ for destination trust = 0.213					
R^2^ for halal food availability = 0.843		β _halal food performance – trust & attachment – retention_ = 0.346 **		β _destination trust_ = 0.643 **	
R^2^ for halal food health/nutrition = 0.758				β _halal food performance_ = 0.590 **	
		β _halal food performance – trust – attachment_ = 0.248 **			
R^2^ for halal food accreditation = 0.880					
R^2^ for halal food cleanness/safety/hygiene = 0.929					

Note1. Goodness-of-fit statistics for the structural model (higher-order framework): χ^2^ = 283.934, *df* = 80, *p* < 0.001, χ^2^/*df* = 3.549, RMSEA = 0.089, CFI = 0.943, IFI = 0.944, TLI = 0.926, * *p* < 0.05, ** *p* < 0.01.

**Table 4:** Baseline and invariance model assessment results.

Paths	High Group of Halal-Friendly Destination Image (*n* = 151)		Low Group of Halal-Friendly Destination Image (*n* = 175)		Baseline Model (Freely Estimated)	Nested Model
	Coefficients	*t*-Values	Coefficients	*t*-Values		(Constrained to Be Equal)
Halal food performance → Trust	0.312 **	3.418	0.227 **	2.747	χ^2^ (168) = 376.699	χ^2^ (169) = 380.863 ^a^
Halal food performance → Attachment	0.105	1.191	0.084	0.886	χ^2^ (168) = 376.699	χ^2^ (169) = 376.985 ^b^
Chi-square difference test:						
^a^ Δχ^2^ (1) = 4.164, *p* < 0.05 (H7a—supported)						
^b^ Δχ^2^ (1) = 0.286, *p* > 0.05 (H7b—not supported)						

Note. Goodness-of-fit statistics for the baseline model: χ^2^ = 376.699, *df* = 168, *p* < 0.001, χ^2^/*df* = 2.242, RMSEA = 0.062, CFI = 0.926, IFI = 0.927, TLI = 0.907, ** *p* < 0.01.

## Data Availability

The dataset used in this research are available upon request from the corresponding author. The data are not publicly available due to restrictions i.e., privacy or ethical.

## Appendix A

**Halal Food Performance** [2,11,12,17]
**Halal Food Availability Factor**
Availability of halal food attracted me to visit tourist places.
Halal food and beverage are served in restaurants and outlets in tourist sites/places.
**Halal Food Health/Nutrition Factor**
Halal foods served in restaurants are healthy and nutritious.
Halal foods that are available in tourist sites/places are healthy and nutritious
**Halal Food Accreditation Factor**
Halal food providers in tourist sites are accredited with halal certification.
Halal food outlets/restaurants in tourist sites clearly display a halal logo.
**Halal Food Cleanness/Safety/Hygiene Factor**
Halal food and beverage offered in tourist sites/places were clean, safe, and hygienic.
Halal foods served in restaurants are clean, safe, and hygienic.
**Destination Trust** [12,29]
I think Korea as a halal-friendly destination is reliable.
I have confidence in Korea as a halal-friendly destination.
I think that Korea as a halal-friendly destination has high integrity.
**Destination Attachment** [16,37]
I like Korea more than other tourist destinations.
I feel emotionally attached to Korea as a tourist destination.
**Halal-friendly Destination Image** [45]
My overall image of Korea as a Halal-friendly destination is positive.
The overall image I have of Korea as a Halal-friendly destination is favorable.
Overall, I have a good image of Korea as a Halal-friendly destination.
**Muslim Traveler Retention** [49]
I am willing to revisit Korea in the near future.
Korea as a Halal family-friendly place will be my first choice when it comes to choosing a destination.
